# Validity of physical activity monitors for assessing lower intensity activity in adults

**DOI:** 10.1186/s12966-014-0119-7

**Published:** 2014-09-28

**Authors:** M Andrés Calabró, Jung-Min Lee, Pedro F Saint-Maurice, Hyelim Yoo, Gregory J Welk

**Affiliations:** Department of Kinesiology, Iowa State University, 283 Forker Building, Ames, IA 50011 USA; School of Health, Physical Education and Recreation, University of Nebraska-Omaha, 6001 Dodge street, Omaha, NE 68118 USA; School of Kinesiology and Recreation, Illinois State University, 100 N University St, Normal, IL 61761 USA; School of Psychology – CIPsi, University of Minho, Campus de Gualtar, Braga, 4710-057 Portugal

**Keywords:** Accelerometer, Activity monitor, Portable metabolic analyzer, Light activity

## Abstract

**Background:**

Accelerometers can provide accurate estimates of moderate-to-vigorous physical activity (MVPA). However, one of the limitations of these instruments is the inability to capture light activity within an acceptable range of error. The purpose of the present study was to determine the validity of different activity monitors for estimating energy expenditure (EE) of light intensity, semi-structured activities.

**Methods:**

Forty healthy participants wore a SenseWear Pro3 Armband (SWA, v.6.1), the SenseWear Mini, the Actiheart, ActiGraph, and ActivPAL monitors, while being monitored with a portable indirect calorimetry (IC). Participants engaged in a variety of low intensity activities but no formalized scripts or protocols were used during these periods.

**Results:**

The Mini and SWA overestimated total EE on average by 1.0% and 4.0%, respectively, while the AH, the GT3X, and the AP underestimated total EE on average by 7.8%, 25.5%, and 22.2%, respectively. The pattern-recognition monitors yielded non-significant differences in EE estimates during the semi-structured period (p = 0.66, p = 0.27, and p = 0.21 for the Mini, SWA, and AH, respectively).

**Conclusions:**

The SenseWear Mini provided more accurate estimates of EE during light to moderate intensity semi-structured activities compared to other activity monitors. This monitor should be considered when there is interest in tracking low intensity activities in groups of individuals.

## Background

The importance of regular physical activity for good health is now well established and well documented (US Physical Activity Guidelines). Public health recommendations have focused on moderate or vigorous activity but recent research shows that time spent in sedentary activities alone may contribute toward cardiometabolic disease risk and the risk of excess weight gain [1,2]. Studies have also demonstrated health benefits associated with the accumulation of light intensity activity [3-5]. These findings highlight the importance of developing better ways to assess light activity, as well as moderate and vigorous activity.

Accelerometry-based activity monitors have proven effective for evaluating locomotor activities but have shown limitations assessing the energy cost of light intensities activities of daily living. Multi-sensor activity monitors and pattern-recognition monitors that integrate physiological data (i.e., heart rate, body temperature, and heat flux) with accelerometers to estimate energy expenditure (EE), offer potential for improving estimates of lower intensity activities. Previous studies have shown that pattern-recognition monitors are capable of more precise determinations of EE (kcal · min^−1^) and levels of PA compared to commonly used accelerometers [6,7].

In a previous study by Berntsen and colleagues [8], researchers assessed the ability of a variety of activity monitors (i.e., SenseWear Pro_2_ Armband, ActiGraph, ikcal, and ActiReg®) to determine the time spent in moderate and vigorous physical activity (MVPA), and TEE, compared to indirect calorimetry (IC), during 120 minutes of unstructured free-living activity. In the study, some monitors performed better than others estimating the time spent in MVPA obtained from indirect calorimetry. The ActiGraph and the SenseWear Pro_2_ Armband overestimated average minutes of MVPA by 2.5% and 2.9%, respectively, while the ikcal and the ActiReg® underestimated average minutes of MVPA by 11.6% and 98.7%, respectively. The ikcal (5%) and SenseWear Armband Pro_2_ (9%) showed lower estimation errors in TEE compared to the ActiGraph (15%) and ActiReg® (21%). Finally, researchers reported a substantial variation in recorded MVPA time and EE estimate, relative to a criterion measure (i.e., indirect calorimetry) among the physical activity monitors assessed.

To our knowledge, no previous studies have compared the ability of accelerometers and multi-sensor activity monitors to assess lower intensity activities of daily living using a portable metabolic analyzer as the criterion measure. The purpose of the present study was to determine the validity of five commercially available activity monitors regardless of posture allocations, including both pattern-recognition monitors and commonly used accelerometers, for estimating EE of lower intensity (Light) activities (1.5-2.9 METS). Light activity represents the largest contribution to the total daily activity since it captures the primary activities of daily living. Determining the accuracy of monitors for assessment of light activity is important for advancing public health research on physical activity and for assessment of total daily energy expenditure.

## Methods

### Participants

Forty healthy men (n = 21) and women (n = 19) volunteered to participate in the study. Approval from the Institutional Review Board was obtained before the beginning of the study. Participants became aware of the procedures and purposes of the study before signing the informed consent document.

#### Instruments

##### Oxycon Mobile 5.0 (OM)

The OM *(Viasys Healthcare Inc, Yorba Linda, CA)* is portable metabolic analyzer that allows measuring oxygen consumption under free-living conditions, and was utilized in this study as the criterion measure. In a recent validation study, the OM provided similar metabolic parameters (VE, VO_2_, and VCO_2_) compared to the Douglas bag method. Mean differences reported in the study were in all cases lower than 5% [9] and Intraclass correlations ranged between 0.798 and 0.925. Expired gases were collected using Hans Rudolph masks *(Hans Rudolf, Inc., Kansas City, MO)*. Volume and gas calibrations were performed before each trial following manufacturer’s instruction.

##### SenseWear Pro_3_ Armband (SWA, Model: 908901PROD2)

The SWA *(BodyMedia Inc., Pittsburgh, PA, USA)* is a wireless multi-sensor activity monitor that is worn on the upper arm over the triceps muscle. The SWA combines accelerometer data with other heat-related sensors to assess physical activity and EE. The SenseWear software estimates EE for each minute of data using complex pattern-recognition algorithms. A Naive Bayes classifier matches the sensor data to the activity class that best describes the current minute. The different activity classes include: walking, running, stationary bike, road bike, rest, resistance exercise, and other activity. Each activity class is linked to a linear regression model mapping the sensor values and body parameters to EE. Separate regression models are utilized for participants of 18 years of age or younger, and for those older than 18 years. Kilocalories and metabolic equivalents (METs) are converted using the equation METs = kcal/hour/kg. The inputs to the Naive Bayes classifier and the regression models include the data recorded in the armband and derived inputs such as the standard deviation of the data over a number of minutes before and after the minute in question (Personal communication, June 2012).

A number of studies [10-12] have demonstrated that the SWA provides valid estimates of EE, but less is known about how it performs for specific intensities or types of movements. In the present study, the version of the software utilized was the SenseWear Professional Software v6.1 (algorithm v2.2.3).

##### SenseWear Mini Armband (Mini, Model:MF-SW)

The Mini *(BodyMedia Inc., Pittsburgh, PA)* is a newer and smaller version of the SWA. The Mini works in a similar manner but includes a triaxial accelerometer instead of a two-axis accelerometer. The Mini files were processed using the SenseWear Professional Software v7.0 (algorithm v2.2.4). Additional information about the Mini monitor can be obtained elsewhere [12].

##### Actiheart Monitor (AH)

The AH (Cambridge Nurotechnology, Cambridge, UK) uses heart rate and a movement sensor, producing a compact ambulatory device equipped with an omnidirectional accelerometer and ECG signal processor. Previous studies support the utility of branched-equation modeling for improving estimation of physical activity energy expenditure in the AH [13,14]. Validation studies have demonstrated acceptable validity and reliability for measuring EE during treadmill walking and running in adults and children, and sedentary, light- and moderate-intensity PAs in adults. In the current study, we utilized the combined activity and HR algorithm available in the device’s software (version 4.0.3.2), as it has been shown to perform well in both low to moderate and moderate to vigorous physical activity intensities [13].

##### ActiGraph GT3X (GT3X)

The GT3X (ActiGraph, Pensacola, FL) is the most commonly used accelerometer for the assessment of physical activity under free-living conditions. It has been recently utilized in a subsample of the National Health and Nutrition Examination Survey (NHANES) to provide objective estimates of physical activity [15]. It has also been included in a recent study describing best practice recommendations for using accelerometers in a physical activity intervention trial [16]. The GT3X is the latest version of the ActiGraph accelerometers and includes a tri-axial accelerometer. Data was collected in minute-epochs.

Following manufacturer recommendations, when the GT3X counts exceeded or equal to 1952 per minute, the following equation published by Freedson et al. [17] was applied to estimate EE: *Kcals/min = 0.00094 × vertical counts/minute + 0.1346 × body mass (kg) – 7.37418.* If the GT3X counts were less or equal to 1952 per minute, the Work-Energy Theorem formula was applied to estimate EE: *Kcals/min = 0.0000191 × counts/minute × body mass (kg).*

##### ActivPAL (AP)

The AP (PAL Technologies Ltd, Glasgow, UK) is a small (7 mm thick) and light (20 g) monitor that, worn on the right thigh, measures acceleration using a piezo-resistive uni-axial accelerometer, that produces a signal related to thigh inclination. The AP is primarily designed to quantify posture allocation and the intensity of an individual’s activity using thigh inclination. Furthermore, the AP software (version 5.8.3.4) allows for classification of activities into periods of sitting/lying, standing, using EE values of 1.25 and 1.4 METs, respectively. In addition, the AP also allows for estimation of EE associated with stepping cadence (i.e., speed), using a pre-defined prediction algorithm (i.e., MET · h = (1.4 × activity duration (hours)) + (4 – 1.4) × (steps per minutes/120) × activity duration (hours)). The AP has been previously validated for posture classification [10,18] and for steps measured at different speeds [17,19] but the AP does not provide a continuous measure of intensity of activities. To our knowledge, no previous study has reported on the validity of the AP to measure EE under free-living conditions.

### Procedures

Participants reported to the laboratory on their scheduled day of testing and were instructed of the characteristics of the study before signing informed consent and health history documents. Measures of standing height, body mass and percentage of body fat were obtained with participants in light clothes and barefooted. Standing height was measured to the nearest 0.1 cm with the use of a wall mounted Harpenden stadiometer (Harpenden, London, UK) and body mass was measured on an electronic scale (Seca 770) to the nearest 0.1 kg. Body mass index (BMI) was calculated as weight (kg)/(m^2^). Percentage of body fat was assessed using a handheld Bioimpedence Analysis device (Omron, Shelton, Connecticut, USA).

Following anthropometric measurements, the participants were fitted the portable metabolic analyzer and the 5 activity monitors. All the instruments were synchronized prior to the measurement. The study was included as part of a larger calibration study lasting 120 minutes, including 60 minutes of structured activities (i.e., stationary biking, walking/running on a treadmill, road biking, elliptical machine, and stair stepper machine) and 60 minutes of unstructured free living movement of light intensity. The semi-structure measurement periods were of various durations (5, 10, 10, 10, and 25-minute intervals) and included unstructured periods spent sitting, walking, standing, stair climbing or light movements. Because the monitors were not developed to detect point estimates for specific physical activities only the free living periods were analyzed in the present study. Additional resting metabolic rate (RMR) measurements were performed in a subsample of participants (24/40 participants) using the same metabolic analyzer, after a 10-hour fast, during the morning of a different scheduled day, and following previously published guidelines [20]. In addition, REE values were estimated for each participant using the REE Mifflin predictive equation for adults [21]. The equation appears to accurately estimate REE in adults [22].

### Data analyses

Data from the OM and the activity monitors were processed on a minute-by-minute basis and merged by time. All statistical analyses were performed using IBM SPSS Statistics 19 software (IBM Corporation, Somers, NY- USA). Paired *t-*tests were used to determine differences between the mean values obtained with the monitors and the portable metabolic analyzer. Bland-Altman plots [23] with corresponding 95% limits of agreement and fitted lines (from regression analyses between mean and difference (upper minus lower limit of agreement)) with their corresponding parameters (i.e., intercept, slope, and respective standard errors (SE)) were presented. A fitted line that provides a slope of 0 and an intercept of 0 exemplifies perfect agreement on average. Agreement throughout this study was indicative of an average estimate of error at a specific energy intensity level and therefore, did not account for proportional bias. In order to test overall associations between measures, Pearson product–moment correlations were computed for TEE. The statistical analyses were conducted at 95% confidence level, and the statistical level was set at *P* < 0.05.

Metabolic equivalents (METs) were calculated from the EE values obtain from the OM and each monitor EE values as the quotient of the energy cost at each minute and each participant’s REE. Accumulation of sedentary (1.0-1.4 METs), light (1.5-2.9 METs) and moderate (3.0-5.9 METs) intensity METs were plotted to compare between monitors (MET-minutes). Classification accuracy of the different monitors was examined using absolute agreement, sensitivity (Se) and specificity (Sp), and kappa values. These indicators examined the extent to what monitors classified correctly (using the OM as the reference measure) average minute-by-minute activity (i.e., 60 minutes) as being sedentary, light, or moderate.

## Results

The sample characteristics were summarized in Table 1. Participant’s age ranged between 18–53 years. The sample was ethnically diverse (42.5% Caucasian, 32.5% Asian, 20.0% Hispanics and 5.0% African-Americans). The participants also varied in body mass index (ranged from 17.8 to 29.0) and percentage of body fat (ranged from 3.0% to 42.2%). During testing, OM data from one participant was lost due to problems with the metabolic analyzer. In addition, AH data was lost from two participants due to lack of heart rate signal detection, and data from two participants were lost due to initialization problems with the AP monitor. Therefore, the final sample included data from 35 participants.

**Table 1:** **Sample characteristics (Mean ± SD)**

	**N**	**Age (years)**	**Height (cm)**	**Weight (kg)**	**BMI** **(kg/m** ^**2**^ **)**	**RMR** **(kcal/day)**
**Males**	21	26.9 ± 4.8	178.5 ± 8.0	76.0 ± 9.6	23.9 ± 3.0	1550.6 ± 163.8
**Females**	19	27.9 ± 8.4	165.8 ± 6.5	59.8 ± 9.0	21.7 ± 2.9	1997.2 ± 389.8
**All**	40	27.4 ± 6.7	172.4 ± 9.7	68.3 ± 12.3	22.9 ± 3.1	1738.1 ± 456.3

### Total energy expenditure (TEE)

Total EE values (means ± SD) were 188.3 ± 51.7, 190.0 ± 51.0, 195.6 ± 48.0, 173.6 ± 56.4, 140.2 ± 76.1, 146.4 ± 43.3 kcal for OM, Mini, SWA, AH, GT3X, and AP, respectively. The multi-sensor monitors (Mini, SWA, and AH) yielded non-significant differences in EE estimates during the semi-structured period, compared to the OM (p = 0.66, p = 0.27 and p = 0.21 for the Mini, SWA, and AH, respectively). In contrast, the accelerometry-based activity monitors (GT3X and the AP) were found to significantly underestimate EE on average when compared to OM (p < 0.001). The Mini and SWA overestimated total EE on average by 0.9% and 3.9%, respectively, while the AH, the GT3X and the AP underestimated total EE on average by 7.8%, 25.5% and 22.2%, respectively. In addition, the absolute error rates (computed as the average absolute value of the individual errors) for each monitor were on average 9.5%, 14.1%, 27.6%, 30.5% and 23.6%, for the Mini, SWA, AH, GT3X and AP, respectively. Pearson product–moment correlations between monitors and OM were r = 0.89 (lower 0.80; upper 0.94), r = 0.70 (lower 0.49; upper 0.83), r = 0.21 (lower −0.10; upper 0.489), r = 0.80 (lower 0.65; upper 0.89) and r = 0.87 (lower 0.76; upper 0.93) for the Mini, the SWA, the AH, GT3X, and AP, respectively.

**Figure 1:**
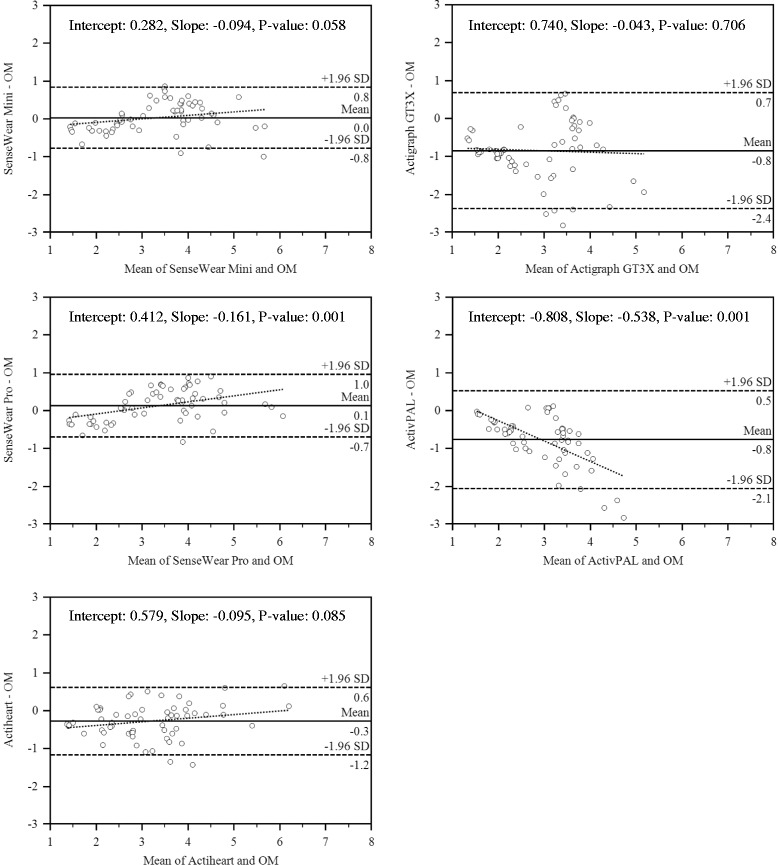
**Bland-Altman plots for the monitors.**

Bland-Altman plots (Figure 1) analyses showed the distribution of error and assist with testing for proportional systematic bias in the estimates. The plots show the residuals of the various EE estimates on the y-axis (OM – estimates) relative to the mean of two methods (x-axis). The plots revealed the narrowest 95% limits of agreement for the Mini (difference = 1.6 METs) and slightly higher values for the SWA (difference = 1.7 METs), and AH (difference = 1.8 METs). Values were higher for the GT3X (difference = 3.1 METs) and the AP (difference = 2.6 METs). A tighter clustering of data points about the mean for Mini, SWA, and AH and less overall error were observed, compared with GT3X and AP. The slopes for the fitted line were not significant for Mini (slope: −0.094, SE: 0.402, *P*: 0.058), AH (slope: −0.095, SE: 0.446, *P*: 0.85), and GT3X (slope:-0.043, SE: 0.826, *P*: 0.70). This suggests no significant patterns of proportional systematic bias with these monitors. However, significant variation in differences across average values were observed for AP (slope:-0.538, SE: 0.501, *P*: 0.001) and SWA (slope:-0.161, SE: 0.382, *P*: 0.001). For the AP, there was a strong increasing amount of underestimation at higher levels of EE; for all other monitors was the opposite (either no difference or a tendency to overestimate relative to OM with increasing average values).

### Energy expenditure during sedentary, light and moderate intensities

**Figure 2:**
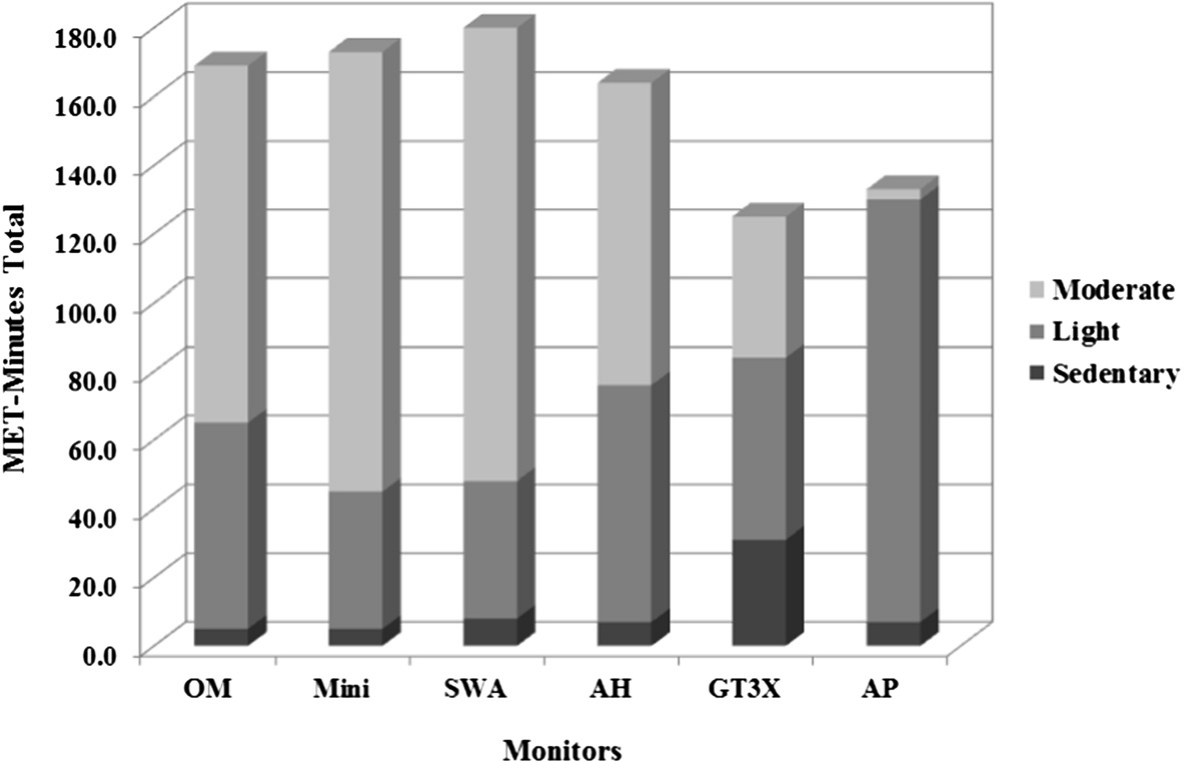
**Comparisons by activity intensity (METS-minutes total).** Abbreviations: Oxycon Mobile, OM; SenseWear Mini Armband, Mini; SenseWear Pro3 Armband, SWA; Actiheart, AH; Actigraph® GT3X, GT3X; ActivPAL, AP.

Comparisons of mean total METs by intensity for each monitor are provided in Figure 2. On average, according to the OM values, participants spent 4 minutes (6.7%) in sedentary intensity (1.0-1.4 METs), 28 minutes (46.7%) in light intensity activity (1.5-2.9 METs) and 28 minutes (46.7%) in moderate intensity activity (3–5.9 METs). Absolute percent agreement ranged from 51.1% to 91.1% and was higher during sedentary activities. Average percent agreement for the Mini, SWA and AH exceeded 85% while for GT3X and AP were equal to 51.1% and 68.9%, respectively. Sensitivity analyses indicated that monitors performed better during sedentary activities but findings in light and moderate were mixed. Only the AH (Se = 0.89, 95% CI: 0.78, 1.00) and AP (Se = 0.93, 95% CI: 0.83, 1.00) monitors had sensitivity values greater than 0.80 during light activities. Sensitivity values for moderate intensity activities exceeded 0.80 for all the monitors except for the GT3X (Se = 0.29, 95% CI: 0.12, 0.45) and the AP (Se = 0.07, 95% CI: 0.00, 0.17) monitors (Table 2). Parallel analysis for Specificity also resulted in better indicators for sedentary and moderate activities. Specificity values exceeded 0.80 for all monitors except GT3X during sedentary activities (Sp = 0.64, 95% CI: 0.52, 0.77). The GT3X (Sp = 0.38, 95% CI: 0.21, 0.54) and the AP (Sp = 0.19, 95% CI: 0.05, 0.32) specificity values were lower than 0.40 during light activities. All the monitors had specificity values for moderate activities that were 0.78 or higher (Table 2). Classification agreement when assessed by kappa values was only greater than 0.50 for the Mini (sedentary, light, and moderate activities), SWA (moderate activities), and AH (light and moderate activities) monitors (Table 3).

**Table 2:** **Sensitivity (Se) and specificity (Sp) for assessed minutes of sedentary, light and moderate intensity by monitor**

	**Sedentary**		**Light**		**MVPA**	
	**Se**	**Sp**	**Se**	**Sp**	**Se**	**Sp**
Mini	1.00 (1.00, 1.00)	0.98 (0.95, 1.00)	0.71 (0.55, 0.88)	0.97 (0.91, 1.00)	0.96 (0.90, 1.00)	0.78 (0.64, 0.92)
SWA	1.00 (1.00, 1.00)	0.95 (0.89, 1.00)	0.64 (0.47, 0.82)	1.00 (1.00, 1.00)	1.00 (1.00, 1.00)	0.78 (0.64, 0.92)
AH	1.00 (1.00, 1.00)	0.96 (0.92, 1.00)	0.89 (0.78, 1.00)	0.84 (0.72, 0.97)	0.82 (0.68, 0.96)	0.97 (0.91, 1.00)
GT3X	1.00 (1.00, 1.00)	0.64 (0.52, 0.77)	0.14 (0.01, 0.27)	0.38 (0.21, 0.54)	0.29 (0.12, 0.45)	0.88 (0.76, 0.99)
AP	1.00 (1.00, 1.00)	0.96 (0.92, 1.00)	0.93 (0.83, 1.00)	0.19 (0.05, 0.32)	0.07 (0.00, 0.17)	1.00 (1.00, 1.00)

*Abbreviations: Oxycon Mobile, OM; SenseWear Mini Armband, Mini; SenseWear Pro3 Armband, SWA; Actiheart, AH; Actigraph® GT3X, GT3X; ActivPAL, AP.*

**Table 3:** **Kappa values for OM and activity monitor minutes of sedentary, light and moderate activity**

	**Kappa**		
	**Sedentary**	**Light**	**Moderate**
Mini	0.88 (0.65, 1.00)	0.69 (0.51, 0.87)	0.74 (0.57, 0.90)
SWA	0.70 (0.39, 1.00)	0.66 (0.48, 0.84)	0.77 (0.61, 0.93)
AH	0.78 (0.49, 1.00)	0.73 (0.56, 0.91)	0.80 (0.64, 0.95)
GT3X	0.19 (0.02, 0.36)	−0.49 (−0.69, −0.27)	0.17 (−0.04, 0.38)
AP	0.78 (0.49, 1.00)	0.11 (−0.05, 0.27)	0.08 (−0.03, 0.18)

*Abbreviations: Oxycon Mobile, OM; SenseWear Mini Armband, Mini; SenseWear Pro3 Armband, SWA; Actiheart, AH; Actigraph® GT3X, GT3X; ActivPAL, AP.*

## Discussion

The aim of the present study was to determine the validity of five commercially available activity monitors, including pattern-recognition monitors and commonly used accelerometry-based activity monitors, for estimating energy expenditure of lower intensity activities of daily living. In the study, the Mini, SWA, and AH monitors provided accurate estimates of energy expenditure during lower intensity activities of daily living. The results demonstrated that the accelerometry-based activity monitors (GTX3 and AP) did not perform as well as more advanced pattern-recognition monitors.

Studies have shown that lower intensity activities are difficult to detect and measure due to its intermittent and arrhythmic nature [13,24]. These activities of daily living are key contributors to an individual’s TEE so it is important to evaluate the ability of monitors to assess the energy cost of these activities. The comparison of TEE estimates in the current study demonstrated that the multi-sensor monitors appear to have advantages compared to the standard accelerometry-based monitors. The Mini, SWA, and AH monitors each provided accurate group level estimates of TEE when compared to the metabolic analyzer.

The Mini and the SWA provided the most accurate estimates of TEE when compared to the criterion measure from the portable metabolic analyzer (The Mini and SWA slightly overestimated EE by 1% and 4%, respectively). In a previous study by Berntsen and colleagues [8], assessing EE estimation and time in MVPA with 4 different activity monitors, SWA results showed estimation errors in the opposite direction. Researchers reported a 9% underestimation of EE by the SWA (software v5.1) compared to a portable metabolic analyzer. In that study, as in the current study, researchers made direct comparisons between different activity monitors and indirect calorimetry under free-living conditions. However, in that previous study [8], participants engaged in higher intensity PAs (such as: walking briskly, biking, table tennis, running) and used an older version of the software. In a recent study by Johannsen and colleagues [12], the SWA and Mini monitors were validated against the doubly labeled water method during 14 days of monitoring. In the study, researchers reported 4% and <0.1% differences in TEE estimation for the SWA and Mini monitors, respectively. Furthermore, both monitors showed similar (8%) absolute error rates compared to the criterion method. In the present study, the Mini showed a similar absolute error rate value (9.5%), while the SWA’s absolute error was higher (14.1%). Additionally, while Bland-Altman plots did not show systematic bias for the Mini, it did suggest that SWA showed a tendency to overestimate EE, perhaps explaining the contrary results between the current study (4% overestimation) and Berntsen’s study [8] results (9% underestimation). In another study comparing the SWA with the doubly-labeled water method in adults,[25] researchers reported a slight but significant underestimation in TEE by the SWA (~5%), and high individual associations (ICC = 0.81, *P* < 0.01). Berntsen and colleagues [8] also reported high levels of individual agreement between the SWA and the metabolic analyzer values (ICC = 0.73, *P* < 0.01). In concordance with those results, agreement between the Mini and SWA monitors and the OM values were moderate to high in this study (Mini: r = 0.90, *P* < 0.01; SWA: r = 0.70, *P* < 0.01).

The AH monitor also yielded non-significant overall estimates of EE (underestimating TEE by 7.8%). A similar study in normal weight adults [14] reported similar results for estimating PAEE for a variety of common lower intensity household activities (1.9-4.3 METs). Researchers reported a comparable 6.0% underestimation of PAEE by the AH, with total error ranging from −441 to 192 kcals. Crouter et al. [6] reported similar findings about the accuracy of AH’s PAEE estimates during free-living activities. In the study, involving 48 adults (ages 21–69 yrs, BMI range: 17.9-40.6 kg/m^2^), participants performed 18 sedentary, household, and leisure-time activities while wearing a portable metabolic analyzer. The AH’s PAEE estimates differed from the IC estimations by 8% mean error. Mean errors associated with separate HR and activity algorithms were 16% and 66%, respectively. A recent study from Spierer et al. [26] compared EE estimates from the AH monitor and an accelerometry-based monitor (Actical) during low intensity activities, walking and jogging. The 27 participants (mean age: 26.4 ± 7.3 yrs, mean BMI: 23.9 ± 2.9 kg · m^2^) performed 18 sedentary, household, and leisure-time activities while wearing a portable metabolic analyzer. The investigators concluded that the EE estimates from the AH were significantly better than the ones from the accelerometry-based activity monitor during activities of low pelvic acceleration (i.e., card playing, sweeping, lifting weights). On the other hand, the accelerometry-based activity monitor performed better during level walking and level jogging. The low TEE correlation between the AH and the reference method (r = 0.21), could be explained by an average large individual error observed in the current study for the monitor’s estimates (27.6% absolute error). Nevertheless, Bland-Altman plots for the AH monitor appear did not support systematic bias for estimates obtained from this monitor.

In the present study, the GT3X and AP monitors largely underestimated TEE (25.5% and 22.2%, respectively) for lower intensity activities. The monitors consistently underestimated TEE for 89% (GT3X) and 91% [27] of the participants. Similarly, Berntsen et al. [8] reported significant underestimation of TEE for an Actigraph accelerometry-based activity monitor (Model: 7164) during moderate and high intensity activities. In that study, the associations between the monitor and the criterion values were high (ICC = 0.73), in concordance with our current study (r = 0.80). Bland-Altman plots between the GT3X and AP monitors relative to the criterion measure showed large mean errors and high 95% limits of the agreement (3.2 METs/min for GT3X and 2.6 METs/min for AP). The accelerometry-based activity monitors had evidence of proportional systematic bias, with the differences becoming larger for those with higher TEE values. This indicates that any extrapolation of these results to field-based research should be considered carefully since the amount of error will likely depend on the type and duration of activities that individuals tend to be engaged during the day. Taken collectively, these results support the established notion [26] that accelerometry-based activity monitors have limitations for estimating the energy cost of lower intensity activities. However, it is important to note that the AP is primarily designed to detect time in different postures (sitting/lying and standing) rather than EE per se. With regard to the AG, Crouter and colleagues [28] concluded that the complex nature of free living activities cannot be estimated with any single linear models like those built into the AG. The use of the Two-regression model and new sophisticated techniques such as artificial neural networking and Hidden Markov Modeling [23,29,30] have shown utility for improving the accuracy of MET estimates. However, these methods demand multiple features (i.e., signal variability) of the acceleration signal besides acceleration counts (e.g., counts per minute) and are not easy for non-analysts to use.

Another perspective supporting pattern recognition monitors is the observed agreement when examining the overall classification agreement into minutes of sedentary, light and moderate intensity. There was clear evidence showing, improved classification accuracy with multi-sensor monitors compared with the accelerometry-based monitors.

Overall, the balance between Sensitivity (Se) and Specificity (Sp) was higher during sedentary activities. In these analyses, Se relates to the ability of a monitor to correctly detect a sedentary activity while Sp relates to the ability of a monitor to detect non-sedentary activities. These two are inversely related and can result in two different types of errors when using a monitor. The high indices of Se suggested that all the monitors are sensitive to low EE rates. However, the relatively low Sp values for the GT3X indicated that this monitor might overestimate the prevalence of sedentary activities by misclassifying minutes of light activities as being sedentary. Se and Sp reached appropriate levels for light activities for all the multi-sensor monitors, except the SWA where Se was equal to 0.64. The substantially low Se values for the GT3X indicates that light activities will most likely be misclassified when this monitor is used. The low Sp indices for the GT3X and AP also suggest that moderate intensity activities will be classified as being light. Moderate or higher intensity activities were classified with reasonable accuracy but Se values for the GT3X and AP were low. Kappa analyses revealed that the Mini was the only monitor that was in concordance with the OM for all activity intensities however, 95% confidence intervals were wide and therefore, revealed some inconsistency in these estimates. The differences in classification of minutes into sedentary, light and moderate intensities allowed understanding the errors in estimation from each monitor and their impacts in the TEE differences.

In concordance with some of our results, a previous study evaluating the SWA under free-living conditions [27], the SWA showed to provide more accurate MET estimates compared to an accelerometry-based activity monitor, using a well validated multisensory [31] (Intelligent Device for Energy Expenditure and Activity (IDEEA)) as the criterion measure. In that previous study [29], the SWA was concurrently compared with different Actigraph EE estimation equations.

One of the strengths of this study is the use of a portable metabolic system that allows measuring daily living physical activity as they generally occur, and the use of five commercially available activity monitors. The concurrent use of a criterion measure and the different monitors allows for direct comparison between methods in order to understand the advantages and limitations of each instrument under the study conditions.

One of the limitations of the study is the short period of time assessed during the protocol. In the study, the protocol (measured) time was limited by the capability of the portable metabolic analyzer’s battery time. The current study would also benefit if there were more observation EE points (e.g., increased number of activities, larger sample size). This limitation resulted in less precise estimates of error/agreement (illustrated by large confidence intervals for EE error and kappa scores). Another limitation was the utilization of a convenience sample (mostly from kinesiology department) in the study, not allowing for generalization of the results to the general population. Most volunteers who participated were active and lean, possibly different than the average population. Lastly, the impossibility to obtain RMR measurements in all participants was an additional limitation in the study. Estimation formulas could possibly introduce additional error to the comparisons.

## Conclusion

In conclusion, the SenseWear Mini provided more accurate estimates of EE during light to moderate intensity free-living activities included in our study when compared to other activity monitors. The SWA and AH multi-sensor monitors provide accurate group estimates of EE during light and moderate semi-structured intensity activities, but showed larger individual estimation error. On the other hand, the accelerometry-based activity monitors showed larger error for estimation of lower intensity activities of daily living. Future research should focus on assessing lower intensity activities using the newly developed techniques to improve MET estimates of accelerometry-based activity monitors (i.e., artificial neural networking and Hidden Markov Modeling), making direct comparisons to multi-sensor activity monitors.
